# Genetic and Molecular Analysis of Root Hair Development in *Arabis alpina*

**DOI:** 10.3389/fpls.2021.767772

**Published:** 2021-10-15

**Authors:** Mona Mapar, Divykriti Chopra, Lisa Stephan, Andrea Schrader, Hequan Sun, Korbinian Schneeberger, Maria Albani, George Coupland, Martin Hülskamp

**Affiliations:** ^1^Botanical Institute, Biocenter, Cologne University, Cologne, Germany; ^2^Faculty of Biology, LMU Munich, Munich, Germany; ^3^Max Planck Institute for Plant Breeding Research, Cologne, Germany

**Keywords:** root hair, *Arabis alpina*, patterning, morphogenesis, SCN1, R2R3MYB, bHLH

## Abstract

Root hair formation in *Arabidopsis thaliana* is a well-established model system for epidermal patterning and morphogenesis in plants. Over the last decades, many underlying regulatory genes and well-established networks have been identified by thorough genetic and molecular analysis. In this study, we used a forward genetic approach to identify genes involved in root hair development in *Arabis alpina*, a related crucifer species that diverged from *A. thaliana* approximately 26–40 million years ago. We found all root hair mutant classes known in *A. thaliana* and identified orthologous regulatory genes by whole-genome or candidate gene sequencing. Our findings indicate that the gene-phenotype relationships regulating root hair development are largely conserved between *A. thaliana* and *A. alpina*. Concordantly, a detailed analysis of one mutant with multiple hairs originating from one cell suggested that a mutation in the *SUPERCENTIPEDE1* (*SCN1*) gene is causal for the phenotype and that *AaSCN1* is fully functional in *A. thaliana*. Interestingly, we also found differences in the regulation of root hair differentiation and morphogenesis between the species, and a subset of root hair mutants could not be explained by mutations in orthologs of known genes from *A. thaliana*. This analysis provides insight into the conservation and divergence of root hair regulation in the Brassicaceae.

## Introduction

Evolutionary studies of development often aim to understand how changes in the function of genes or gene networks result in phenotypic differences. One approach is to compare gene functions in two species that are sufficiently evolutionarily distant to find differences but close enough to identify orthologous genes. In plants, *Arabidopsis thaliana* serves as an ideal reference system for evolutionary comparisons, due to the rich knowledge of the genetic, molecular, and cell-biological mechanisms underlying various developmental processes. Within the Brassicaceae family, *Arabis alpina* has been established as an additional genetic model system (Koch et al., 2006; Wang et al., 2009, 2011; Poncet et al., 2010). *A. thaliana* and *A. alpina* have an evolutionary distance of 26–40 million years (Koch et al., 2006; Beilstein et al., 2010). The genome of *A. alpina* is fully sequenced (Willing et al., 2015; Jiao et al., 2017), and orthologous genes can be identified by homology and their relative position on the chromosomes (synteny). The exhaustive search for trichome mutants in *A. alpina* and their molecular analysis revealed a similar genetic network as described in *A. thaliana* (Chopra et al., 2014, 2019). However, distinct differences were also found between both Brassicaceae species, in particular for the function of genes involved in trichome patterning (Chopra et al., 2019).

In *A. thaliana*, root hairs form in single-cell files (H-files), which develop above the cleft of two cortical cells. Non-hair files (N-files) are formed over cortical cells (Dolan et al., 1994; Schiefelbein, 2000). The positional information from the underlying cortex cells is mediated by the leucine-rich repeat receptor-like kinase *SCRAMBLED* (*SCM*; Kwak et al., 2005). H-file cells (trichoblasts) and N-file cells (atrichoblasts) differ from early development. Trichoblasts display a higher cell division rate (Berger et al., 1998), reduced cell length (Dolan et al., 1994; Masucci et al., 1996), and denser cytoplasm (Dolan et al., 1994; Galway et al., 1994). In *A. thaliana*, most trichome patterning genes are also involved in root hair patterning (Schiefelbein, 2003; Schellmann et al., 2007). Proteins encoded by the patterning genes include the R2R3MYB transcription factor WEREWOLF (WER; Lee and Schiefelbein, 1999) and the WD40 protein TRANSPARENT TESTA GLABRA 1 (TTG1; Galway et al., 1994) that form a complex with the bHLH proteins GLABRA3 (GL3; Payne et al., 2000) and ENHANCER OF GLABRA 3 (EGL3; Zhang et al., 2003). The activity of this complex is counteracted by the partially redundant R3MYBs CAPRICE (CPC) and TRIPTYCHON (TRY; Wada et al., 1997; Schellmann et al., 2002; Schiefelbein, 2003) and their activator ENHANCER OF TRY AND CPC 1 (ETC1; Simon et al., 2007). Ultimately, the cooperation of these MYB, bHLH, and WD40 genes activates the expression of *GLABRA2* (*GL2*), which encodes a homeodomain transcription factor that suppresses root hair development in non-root hair cells (Masucci et al., 1996).

Root hair development begins with polarized outgrowth at the basal end of the cell (Molendijk et al., 2001). Initially, RHO-RELATED PROTEIN FROM PLANTS (ROP) localizes to the growth site and remains localized at the very tip of the growing root hair during root hair extension (Molendijk et al., 2001). Tip growth involves the condensation of the endoplasmic reticulum (Ridge et al., 1999), F-actin accumulation (Baluška et al., 2000), and microtubules, which are involved in regulating the localization and size of the bulges (Kost et al., 1999). The second phase of root hair formation begins when hairs are about 40μm long (Dolan et al., 1994). It is characterized by localized growth at the tip of the hair (Carol et al., 2005). This process is organized by polarized cytoplasm in the bulge, which mediates localized secretion and cell wall synthesis (Favery et al., 2001). In summary, the major molecular events during tip growth include a tip-focused calcium influx (Wymer et al., 1997), cytoskeleton re-modelling (Geitmann and Emons, 2000), polarized membrane trafficking (Campanoni and Blatt, 2007), and cell wall synthesis (Favery et al., 2001).

Root hair development in *A. alpina* is similar to *A. thaliana* (Chopra et al., 2014, 2019). Early in development two files of morphologically different cells, as judged by their cell length, are formed. However, in contrast to *A. thaliana*, 30–40 percent of cells in N-file positions also form root hairs (Chopra et al., 2014). The phenotypic analysis of *Aattg1* and *Aagl3* revealed that both mutants have excessive root hair production (Chopra et al., 2014, 2019). For *TTG1*, this implies similar functions in *A. alpina* and *A. thaliana*. For *GL3*, however, this finding represents a striking difference to *A. thaliana* where extra hairs are found in *gl3 egl3* double mutants but not in the single mutants (Bernhardt et al., 2003).

In this study, we screened an ethyl methane-sulfonate (EMS)-mutagenized population of *A. alpina* plants for root hair mutants. We uncovered mutants affecting root hair cell patterning and most steps of root hair development. We identified orthologous genes in *A. alpina* by taking into consideration not only the sequence similarity but also the relative position of genes on the chromosomes. Candidate genes were sequenced in the mutants to reveal the gene-phenotype relations in *A. alpina*. These data demonstrate that root hair cell regulation is largely conserved between the two species, but also identify interesting differences.

## Materials and Methods

### Plant Material and Growth Condition

All *A. alpina* mutants were isolated from EMS-mutagenized *A. alpina Pajares* and *pep1-1* populations (Wang et al., 2009; Chopra et al., 2014). The *A. thaliana scn1-3* mutant was described previously (Carol et al., 2005).

For root hair analysis, seeds were surface sterilized with chlorine gas for 3h. Sterilized seeds were sown on full Murashige-Skoog plates (Murashige and Skoog, 1962) w/o sucrose and stratified at 4°C for 5days. Plants were grown vertically for 7days under long-day conditions (16-h light, 8-h darkness) at 21°C.

For whole-genome sequencing, plants were grown on plates as described before and the root phenotypes were confirmed in the M3 generation. Next, the seedlings were transferred to soil for three more weeks. Fresh leaf samples of 1-month-old plants were used for DNA extraction.

### Sequence and Synteny Analysis

*Arabis alpina* gene sequences were obtained from the Genomic resources for *A. alpina* website^1^ and analyzed with CLC DNA Workbench 5.6.1.

For sequence analysis by Sanger sequencing, primers were designed outside the CDS of a given *A. alpina* gene to sequence it in the mutants. Library preparation and whole-genome sequencing were carried out by the Cologne Center for Genomics, and the raw sequence reads were deposited into National Center for Biotechnology Information^2^ under accession number PRJNA745061. Short reads of each mutant sample were aligned to the *A. alpina* reference genome version 5.1 (Jiao et al., 2017) using *Bowtie2* version 2.2.8, which were further processed with SAMtools version 1.4 (Li et al., 2009) to get BAM files. Next, each BAM was provided to SHORE version 0.8 (Ossowski et al., 2008) to call consensus and variants for each mutant sample. SNPs were stringently selected for each mutant with a minimum mutant allele frequency of 0.85 and a coverage of at least 3. Effects of selected mutations on gene integrity were annotated with SHOREmap version 3.0 (Schneeberger et al., 2009; Sun and Schneeberger, 2015).

The EMBL-EBI database (Pfam 32.0^3^; Finn et al., 2016) was used to identify conserved domains.

GBrowse from TAIR 10,^4^ along with the assembled *A. alpina* genome, was used to confirm the synteny of the selected genes regarding conserved order and appearance of the neighboring genes.

### Plasmids and Stable Plant Transformation

*AtSCN1* and *AaSCN1* were amplified by PCR, using *A. thaliana* Col-0 or *A. alpina* Pajares cDNA as templates, and subsequently cloned into pDONOR201 and/or pDONOR207 using the Gateway system (Thermo Fisher Scientific). For construction of 35S::*AtSCN1*-YFP and 35S::*AaSCN1*-YFP, the Gateway destination vector pEXSG-YFP (Feys et al., 2005) was used. For construction of the *Aascn1-1* mutant CDS, site-directed mutagenesis was performed on pDONOR207-*AaSCN1* and *Aascn1-1*-YFP was subsequently cloned into pEXSG-YFP (Feys et al., 2005). Plants were transformed using floral dip (Clough and Bent, 1998).

### Root Hair and Root Epidermal Cell Measurements

The number of root hairs was determined in a 1mm long section as described before (Grierson and Schiefelbein, 2002). The position of H-files was determined on 7-day-old seedlings with respect to the position of underlying cortex cells.

### Staining of Roots

For epidermal cell length analysis, roots were stained with 100mg/ml propidium iodide for 1min and subsequently washed with water. Nitroblue tetrazolium chloride (NBT, Sigma) was used to identify sites of superoxide production (Fryer et al., 2002). Seedlings were covered with NBT solution (0.5mg/ml NBT in 0.1M potassium phosphate pH 7) for 10min.

### Microscopical Methods

Stereo microscopy was carried out with the Leica MZ 16F stereo microscope and the LAS AF software (Leica Microsystems, Heidelberg, Germany). Light and fluorescence microscopy were performed with the Leica DMRA2, DMRB, and DM5000B fluorescence microscope (Leica Microsystems, Heidelberg, Germany). Confocal laser scanning microscopy was carried out with the Leica DM5500 CS Microscope and documented with the TCS-SPE imaging systems (Leica Microsystems, Heidelberg, Germany).

Adobe Photoshop CS4 version 14.0.0 and ImageJ (Fabrice Cordelieres, Institut Curie, Orsay, France) were used for image processing.

### Statistical Analysis

Data were analyzed with Microsoft Excel 2007. Statistical analysis was carried out with OriginPro 8.5 0G SR0 and Microsoft Excel 2010. Significance was tested using a two-sided t test for normally distributed data and one-way ANOVA for data with non-normal distribution.

## Results

### Root Hair Development in *A. alpina*

**Figure 1:**
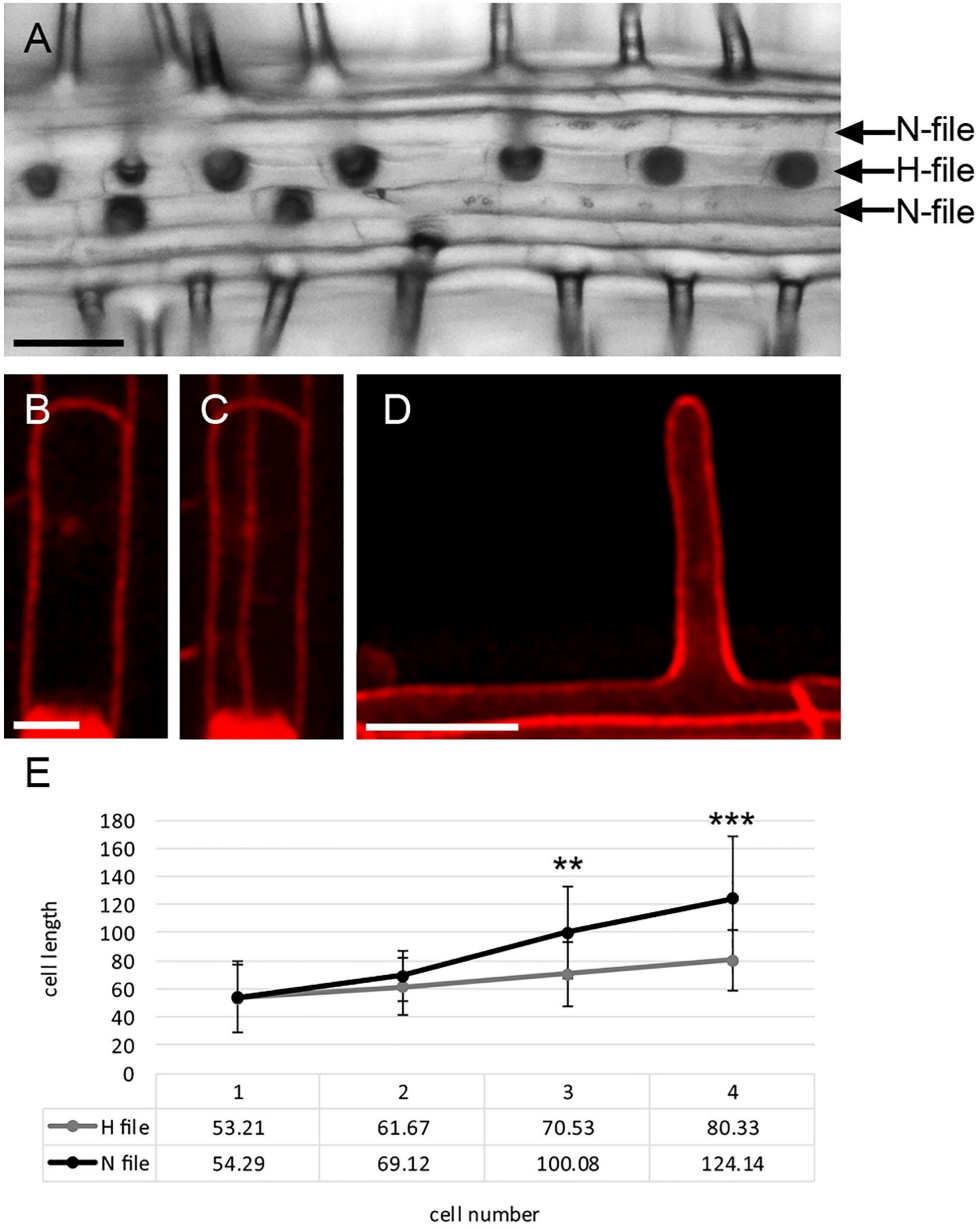
Root hair development in *Arabis alpina*. **(A)** Light microscope picture of the root epidermis. The position of cell files relative to the cortex cells is indicated with the H-position defined to be above the cleft between two underlying cells and the N-position above underlying cells. Scale bar: 50mm. Propidium iodide-stained cell in the H-position: **(B)** the plane of the epidermal cell, **(C)** the plane of the epidermal cell and of the cortex cells. Scale bar: 25mm. **(D)** Propidium iodide-stained root hair growing out at the apical end of the root hair cell. Scale bar: 20mm. **(E)** Quantitative analysis of cell length in N- and H-positions. Cell length is shown for four cells in a cell file with the first cell representing the closest to the tip and the fourth cell being immediately below the first root hair cell showing a bulge. Significance levels were determined at *p*<0.001 (***), *p*<0.01 (**).

Root hair development in *A. alpina* is similar to *A. thaliana*. Root hairs are initiated at the basal end of root epidermal cells, followed by extensive tip growth (Figures 1A,D; Grierson and Schiefelbein, 2002). However, all cell files in *A. alpina* can produce root hairs [(Chopra et al., 2014), Figure 1A]. Nevertheless, trichoblasts and atrichoblasts differ in cell length (Chopra et al., 2014), so it is conceivable that *A. alpina* also produces morphologically distinct H- and N-files in the root epidermis. To substantiate this, root hairs were stained with propidium iodide to identify the H-file position by their position over the cleft of the underlying cortex cells (Figures 1B,C). We measured the length of four consecutive cells in N- and H-files starting immediately below the first cell displaying the formation of a root hair bulge. Cells located close to the root tip show no length difference. More distant cells were about 30% shorter in H-positions as compared to N-positions (Figure 1E), indicating that a morphological difference between these cell types is conserved between the two species.

### Isolation of Root Hair Mutants in *A. alpina*

**Figure 2:**
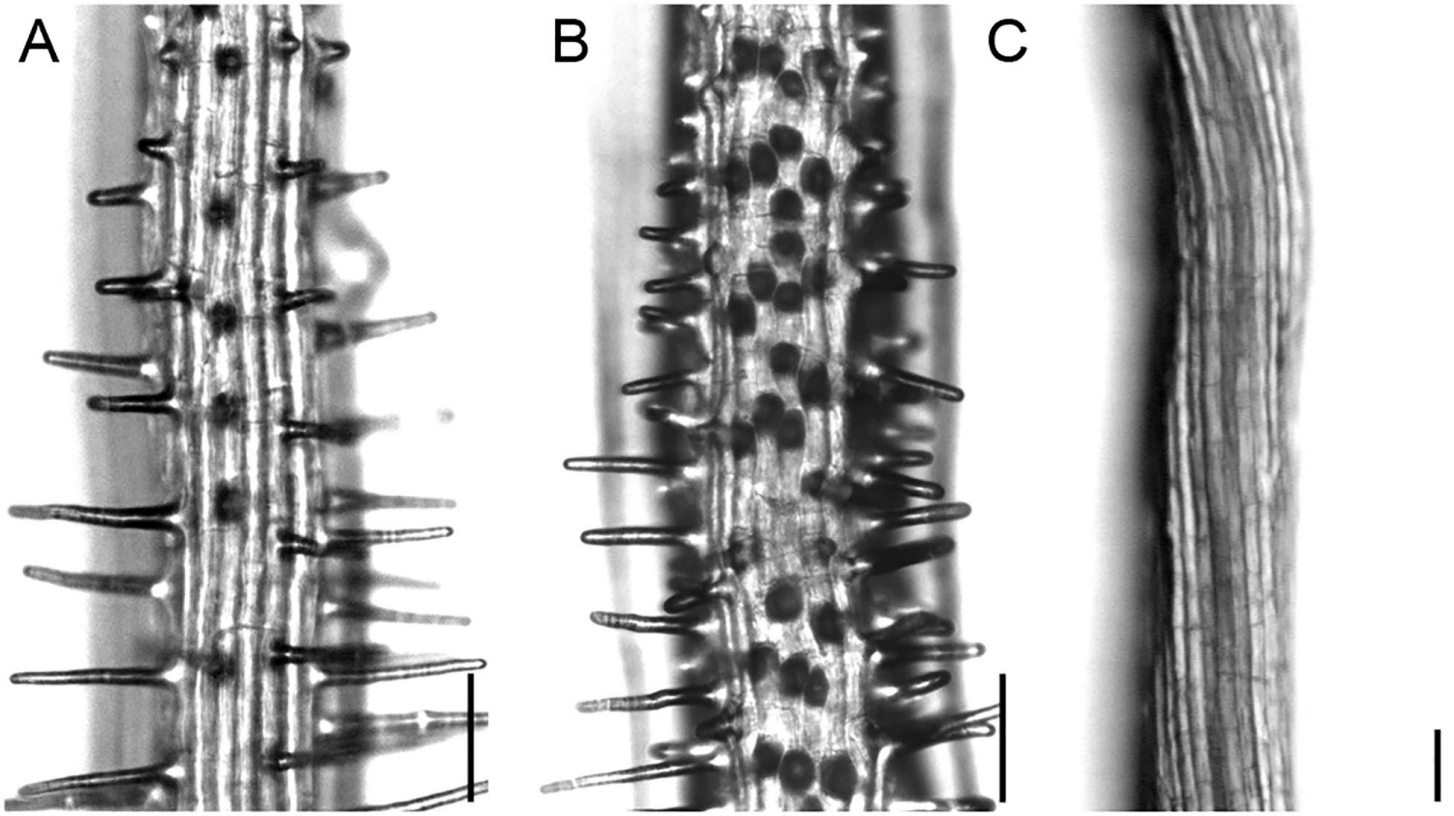
Root hair patterning phenotypes in *A. alpina*. Light microscopic images of **(A)** wild type, **(B)** increased root hair number (line 1,264), **(C)** no root hairs (line 1,101). Scale bar: 100mm.

**Figure 3:**
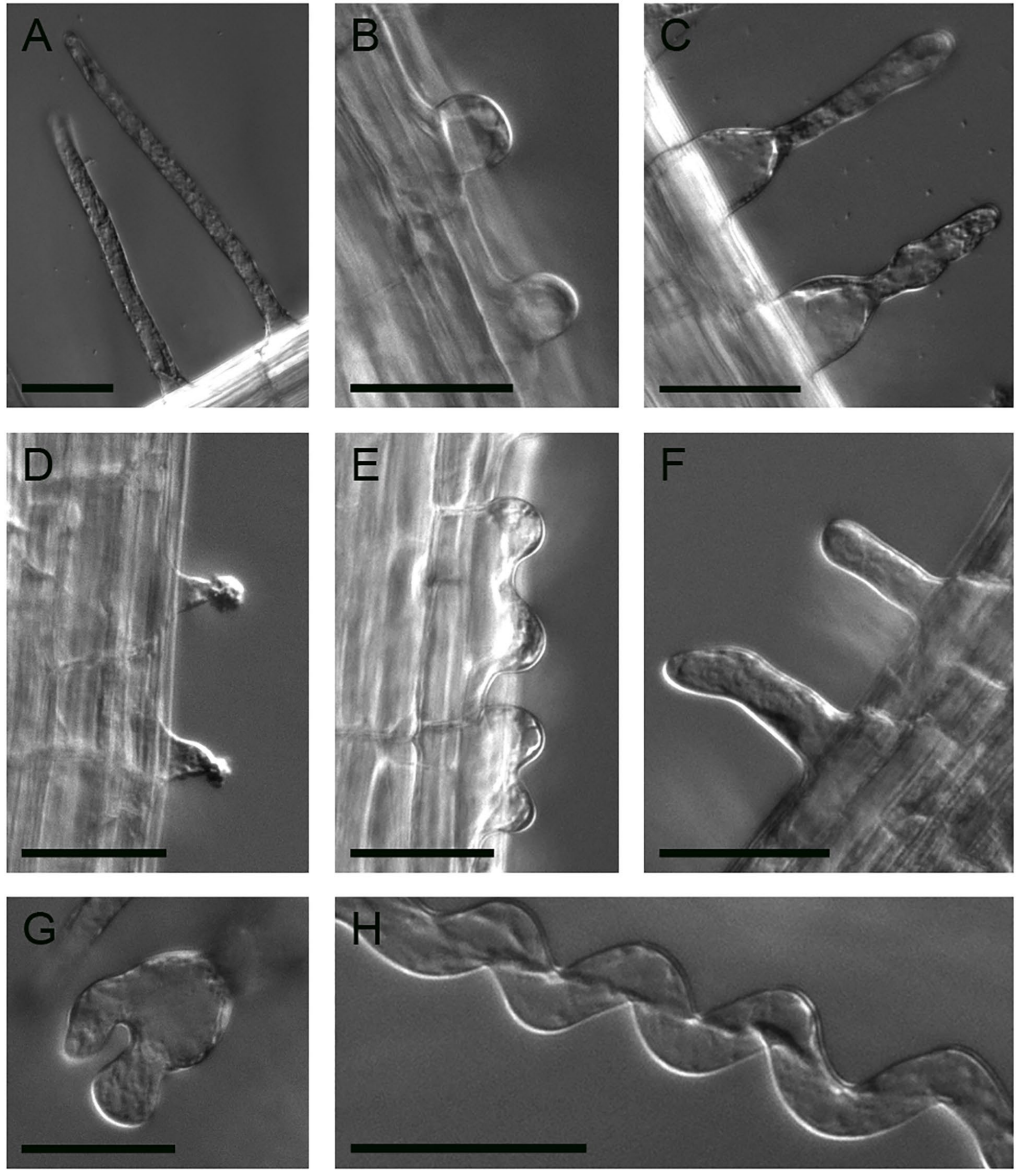
Root hair morphogenesis phenotypes. **(A)** Wild-type root hair. **(B)** Root hair growth stops after bulge formation (line 366). **(C)** Root hairs are swollen at the base (line 1,221). **(D)** Bursting root hairs (line 1,185). **(E)** Multiple outgrowths from each epidermal cell (line 1,384). **(F)** Short root hairs (line 1,091). **(G)** Branched root hairs (line 216). **(H)** Wavy root hairs (line 216). Scale bar: 50μm.

We used a forward genetic approach to define different functional steps during root hair development in *A. alpina*. Two EMS screens were performed in independent populations: one in *Pajares* (Wang et al., 2009), representing 4,205M1 plants, and the other in the *pep1-1* background (Wang et al., 2009; Albani et al., 2012; Bergonzi et al., 2013; Zhou et al., 2021), representing 6,800M1 individuals. Seeds of five M1 plants were pooled, and 50 M2 seedlings from each pool were screened for root hair phenotypes. Forty-five root hair pattern and root hair morphology mutant phenotypes were confirmed in the M3 generation (Table 1). We found two types of patterning mutants: Seven mutants showed more root hairs (Figure 2B), and six had fewer or no root hairs (Figure 2C) compared to the wild type (Figures 2A and 3A). The morphology mutants were divided into six classes. Three lines produced only small bulges (Figure 3B), suggesting that cell elongation is disturbed. In six mutants, hairs initiated from a swelling of the epidermal cell (Figure 3C). Three lines showed a bursting phenotype (Figure 3D). Typically, these root hairs ruptured at the tip soon after initiation. One line produced multiple sites of growth from each epidermal cell (Figure 3E). Sixteen mutants exhibited short hairs (Figure 3F), and three mutants produced branched (Figure 3G) and wavy root hairs (Figure 3H). A similar range of phenotypes has been previously described in *A. thaliana* (Grierson and Schiefelbein, 2002).

**Table 1:** Classes of root hair mutants in *A. alpina*.

	Root hair phenotype	*Pajares*	*pep1-1*	Total
Patterning	More hairs	2	5	7
	Fewer hairs	3	3	6
Morphology	Only bulge	1	2	3
	Swollen hairs	1	5	6
	Bursting hairs	1	2	3
	Multiple hairs	1	0	1
	Short hairs	2	14	16
	Branched/wavy hairs	1	2	3
	Total	12	33	45

### Identification of Root Hair Genes in *A. alpina*

*A. alpina* and *A. thaliana* are both members of the Brassicaceae and therefore relatively closely related (Koch et al., 2006; Beilstein et al., 2010). Hence, we reasoned that most mutant phenotypes found in the *A. alpina* root hair screen are probably caused by mutations in orthologs of genes shown to affect root hair development in *A. thaliana*. In a first step, we selected genes known to be involved in root hair development of *A. thaliana* (Supplementary Table S1). We also included genes involved in trichome development, because of genetic overlap between the two processes, and pollen tube genes, since pollen tubes show tip growth similar to root hairs. In total, we considered 136 *A. thaliana* genes. In a next step, we identified 283 corresponding paralogs or orthologs based on the annotated *A. alpina* genome (Willing et al., 2015; Supplementary Table S1). This way, we constructed a list of candidate *A. alpina* genes that might be impaired in function in our set of root hair mutants.

### Identification of Mutant-Specific Alleles

We then sequenced candidate genes in selected root hair mutants, as previously described for trichome genes in *A. alpina* (Chopra et al., 2014, 2019). However, the high number of candidate genes rendered gene by gene sequencing inefficient. Therefore, we performed whole-genome sequencing of the genomic DNA from different mutants, aiming for an average of 15X fold coverage. Each SNP identified in the genes of interest is listed in Supplementary Table S2 along with the position in the chromosome, the sequence of the reference allele and the mutant allele, the location of exchange regarding the gene model, mutant allele coverage, and mutant allele frequency. Annotated SNPs, stringently selected for each mutant, with a minimum mutant allele frequency of 0.85 and minimum mutant allele coverage of 3, were chosen for further analysis. Next, we correlated SNPs to the phenotype of the respective mutants. As summarized in Supplementary Table S3, 26 mutant-specific alleles were identified. We considered stop codons as mutant-specific alleles, while other mutations were only considered when leading to a non-synonymous amino acid exchange.

#### Root Hair Patterning Mutants

The analysis of the seven mutants with ectopic root hair production revealed one line with a mutation in the *AaTTG1* and six lines with mutations in the *AaGL3* genes (Supplementary Table S3), with similar phenotypes as described previously (Chopra et al., 2014, 2019). None of the mutants with an ectopic root hair phenotype exhibited mutations in *AaWER*, although the Arabidopsis *wer* mutant shows this phenotype. Six mutants exhibited fewer root hairs as compared to the wild type. None of these showed mutations in any of the selected candidate genes compiled in Supplementary Table S1.

#### Root Hair Morphology Mutants

**Figure 4:**
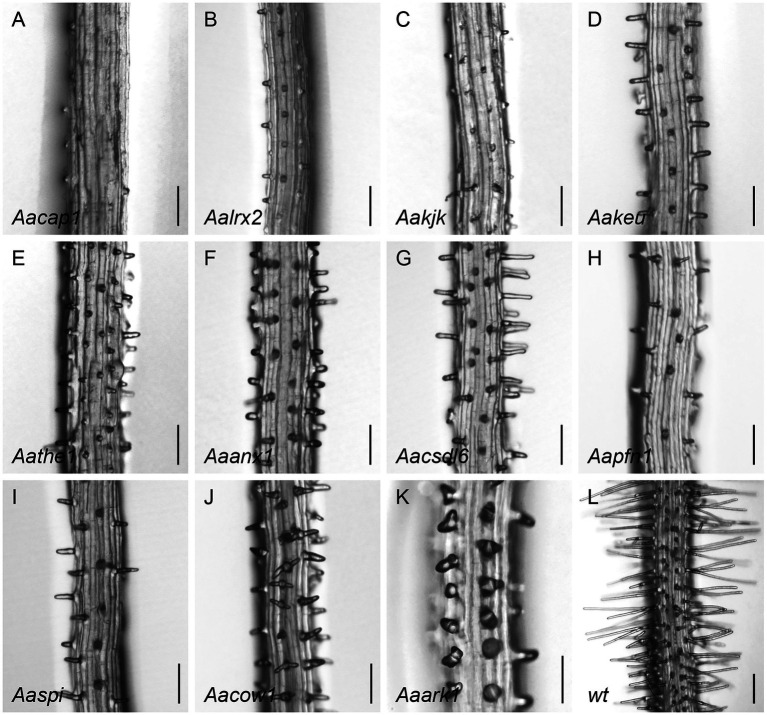
Root hair phenotypes of identified *A. alpina* mutants in **(A)** *[CA2+]CYT-ASSOCIATED PROTEIN KINASE 1* (*CAP1*), **(B)** *LEUCINE-RICH REPEAT/EXTENSIN 2* (*LRX2*), **(C)** *KOJAK* (*KJK*), **(D)** *KEULE* (*KEU*), **(E)** *THESEUS 1* (*THE1*), **(F)** *ANXUR 1* (*ANX1*), **(G)** *CELLULOSE SYNTHASE-LIKE D6* (*CSLD6*), **(H)** *PROFILIN 1* (*PFN1*), **(I)** *SPIRRIG* (*SPI*), **(J)** *CAN OF WORMS1* (*COW1*), **(K)** *ARMADILLO REPEAT-CONTAINING KINESIN 1* (*ARK1*), and of the **(L)** wild type. Scale bar: 250μm.

Among the 38 root hair morphology mutants (Figure 4), we identified 12 genes displaying mutations in our collection of candidate genes (Supplementary Figure S1; Supplementary Table S3).

Sequencing of three mutants showing only small bulges confirmed allele-specific changes in two of them. We found an E to K exchange at position 465 in the *[CA2^+^]CYT-ASSOCIATED PROTEIN KINASE 1* (*CAP1*) gene (Figure 4A; Supplementary Figure S1A). CAP1 is a receptor-like kinase which maintains cytoplasmic Ca2+ gradients and is important for root hair growth in *A. thaliana*. The *Atcap1* mutant displays shorter, slightly malformed hairs (Bai et al., 2014). In the *Aacap1* line, the mutant phenotype appeared slightly stronger as compared to the mutant in *A. thaliana*, as only small bulges without any outgrowth were formed. The second small bulge mutant showed a P to T exchange at position 588 in *LEUCINE-RICH REPEAT/EXTENSIN 2* (*LRX2*; Figure 4B; Supplementary Figure S1B). In *A. thaliana*, LRX2 and its paralog LRX1 act together during root hair elongation such that *Atlrx1* single mutants and *Atlrx1 Atlrx2* double mutants but not *Atlrx2* mutants exhibit aborted, swollen, or branched root hairs (Baumberger et al., 2003).

Sequencing of the six mutants with swollen root hairs revealed no relevant mutations in any of the selected candidate genes.

All three mutant lines showing bursting root hairs carried mutations in the *KOJAK* (*KJK*) gene (Figure 4C; Supplementary Figure S1C). The phenotype is similar to that of *A. thaliana kjk* mutants (Favery et al., 2001), indicating a similar function of KJK in both species. KJK is a cellulose synthase-like protein required for the synthesis of non-cellulose cell wall polysaccharides. In one line, we found a premature STOP codon at a similar position [W (843) to STOP] as in *Atkjk-3* (Favery et al., 2001) and two other alleles presented G (248) to R and T (854) to M substitutions in conserved regions, which are expected to cause severe defects in protein function.

Analysis of 16 short root hair mutants revealed mutations in eight known root hair genes. In one line, we found a mutation in the *KEULE* (*KEU*) gene (Figure 4D; Supplementary Figure S1D), which encodes a Sec1 protein required for cytokinesis (Grierson and Schiefelbein, 2002). The mutation leads to a G (487) to R exchange within the Sec1 domain. Unlike in *A. thaliana* (Grierson and Schiefelbein, 2002), we did not observe swollen root hairs in the *Aakeu* mutant. In addition, we found mutant lines with SNPs in *THESEUS 1* (*THE1*) and *ANXUR 1* (*ANX1*; Hématy et al., 2007; Boisson-Dernier et al., 2009; Miyazaki et al., 2009). Both encode receptor-like tyrosine kinase proteins (Cheung and Wu, 2011). *Aathe1* has a G (614) to R exchange in the tyrosine kinase domain and *Aaanx1* a D (813) to N exchange after the tyrosine kinase domain (Figures 4E,F; Supplementary Figures S1E,F). The respective *A. thaliana* mutants appear to have a stronger phenotype as the root hairs are not only shorter but may collapse or burst (Duan et al., 2010; Cheung and Wu, 2011). We also identified one short root hair line with an E (289) to K exchange in the CELLULOSE SYNTHASE-LIKE D6 (CSLD6) protein (Figure 4G; Supplementary Figure S1G). The members of the CSLD family, including *KJK* (*CSLD3*), are known to be essential for the structural integrity of cell walls of tip-growing cells (Bernal et al., 2008). It is therefore conceivable that AaCSLD6 plays a role in root hair development in *A. alpina*. In one of the short root hair mutants, we found a G (64) to E substitution in *PROFILIN1* (*PFN1*; Figure 4H; Supplementary Figure S1H). PFN is an actin-binding protein involved in the organization of the cytoskeleton which plays a general role in cell elongation in *A. thaliana* (Ramachandran et al., 2000). Similar as in *Aapfn1*, *A. thaliana 35S::antisense PFN 1* lines displayed shorter root hairs (Ramachandran et al., 2000). Two short root hair mutants had STOP codons in *SPIRRIG (SPI)* at positions 2,107 and 1976, similar to *Atspi-4.2* (Saedler et al., 2009; Figure 4I; Supplementary Figure S1I). Four *spi* alleles in *A. alpina* with similar phenotypes have been previously reported (Chopra et al., 2019; Stephan et al., 2021). In four short root hair mutants, we found meaningful SNPs in the *CAN OF WORMS 1* (*COW1*) gene (Grierson et al., 1997; Böhme et al., 2004; Figure 4J; Supplementary Figure S1J). *cow1* hairs in *A. thaliana* are shorter and wider than wild type and occasionally produce two hairs (Grierson et al., 1997; Böhme et al., 2004). Detailed analysis of the four identified *Aacow1* alleles revealed the same range of phenotypes. Mutations in *AaCOW1* included one premature STOP codon at position 505, two splicing site mutations [G (1849) to A and G (1679) to A], resulting in STOP codons at different positions similar to *Atcow1* alleles (Grierson et al., 1997; Böhme et al., 2004). In one allele, we found a P (293) to L substitution in the conserved CRAL-TRIO lipid binding domain (Supplementary Figure S1).

Sequencing of mutants with wavy and branched root hairs revealed STOP codons at positions 979 and 899 in the *ARMADILLO REPEAT-CONTAINING KINESIN 1* (*ARK1*) gene (Yang et al., 2007; Yoo and Blancaflor, 2013; Figure 4K; Supplementary Figure S1K). *ARK1* controls microtubule organization during root hair tip growth. In *Atark1* mutants, fragmentation and random orientation of microtubules results in wavy/spiral and branched root hairs (Yang et al., 2007; Yoo and Blancaflor, 2013; Rishmawi et al., 2014).

The protein sequence analysis of all identified morphology genes showed a high similarity between *A. alpina* and *A. thaliana* (Supplementary Table S4), and synteny is also maintained for these genes between both species (Supplementary Figure S2).

#### Multiple Hairs: Analysis of *AaSCN1* in *A. alpina*

**Figure 5:**
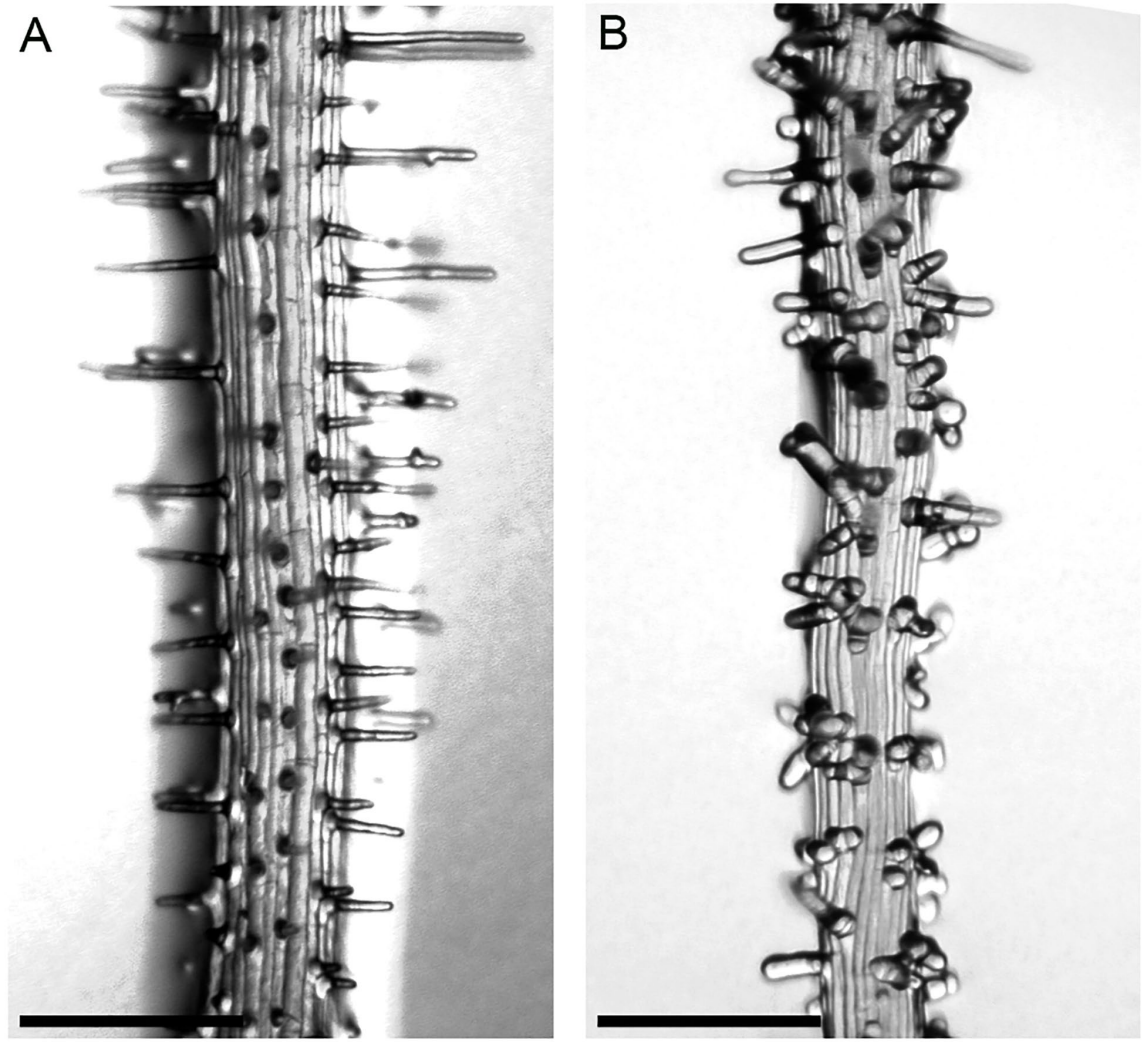
Root hair phenotype of the *Aascn1* mutant. **(A)** Wild type. **(B)** *Aascn1* exhibits multiple bulges initiated from each epidermal cell and multiple growing axes initiated from each bulge. Scale bar: 250μm.

One of the first mutants identified showed multiple bulges that are initiated from each epidermal cell and multiple growing axes initiated from each bulge (Figure 5). This mutant was shown to have a STOP codon in the *SCN1* gene (Carol et al., 2005; Supplementary Figure S1). We decided to analyze this mutant in more detail in parallel to the systematic analysis of the other mutants. *SCN1* encodes a RhoGTPase GDP dissociation inhibitor (RhoGDI), which is involved in localizing the RHD2/AtrbohC NADPH oxidase to the tip of root hairs (Carol et al., 2005). The RHD2/AtrbohC NADPH oxidase is required for ROS production, which in turn is essential for root hair elongation (Foreman et al., 2003). The AaSCN1 protein shows 84 percent similarity to AtSCN1 (Supplementary Table S4), and synteny is also maintained between the two species (Supplementary Figure S2).

**Figure 6:**
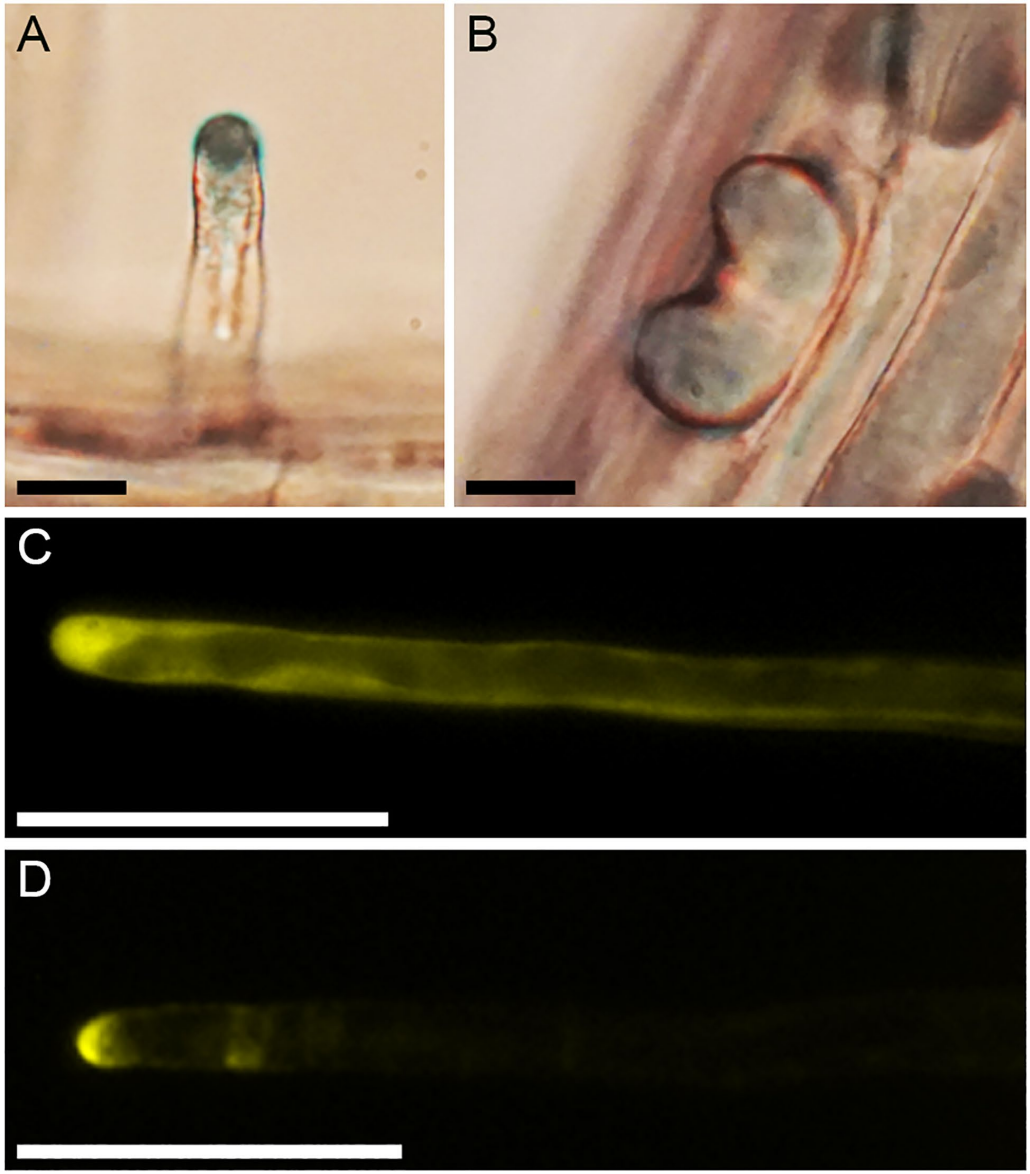
ROS and SCN1 localization in root hairs. NBT staining of **(A)** wild type *A. alpina* and **(B)** *Aascn1-1*. Localization of the SCN1 protein from **(C)** *A. alpina* and **(D)** *Arabidopsis thaliana* in *A. thaliana* Col-0 background. Scale bar: 10μm (A, B) and 50μm (C, D).

To monitor the spatial production of ROS, we used Nitrobluetetrazolium (NBT). NBT is reduced to a blue formazan precipitate in the presence of ROS (Fryer et al., 2002). NBT staining in the root hairs of wild type showed blue staining at one single point of the hair tip in the wild type (Figure 6A). In the *Aascn1-1* mutant, the blue color was not observed in a focal point but in a broader area (Figure 6B). Next, we analyzed the localization of the SCN1 protein from *A. alpina* and *A. thaliana*. The *SCN1* CDS was fused to YFP and expressed in *Col-0* plants under control of the 35S promoter. Two lines for AtSCN1-YFP and two lines for AaSCN1-YFP were analyzed. Both *A. alpina* and *A. thaliana* SCN1-YFP were detected at the tip of growing hairs (Figures 6C,D). However, AaSCN1-YFP was less restricted to the tip than its *A. thaliana* ortholog.

**Figure 7:**
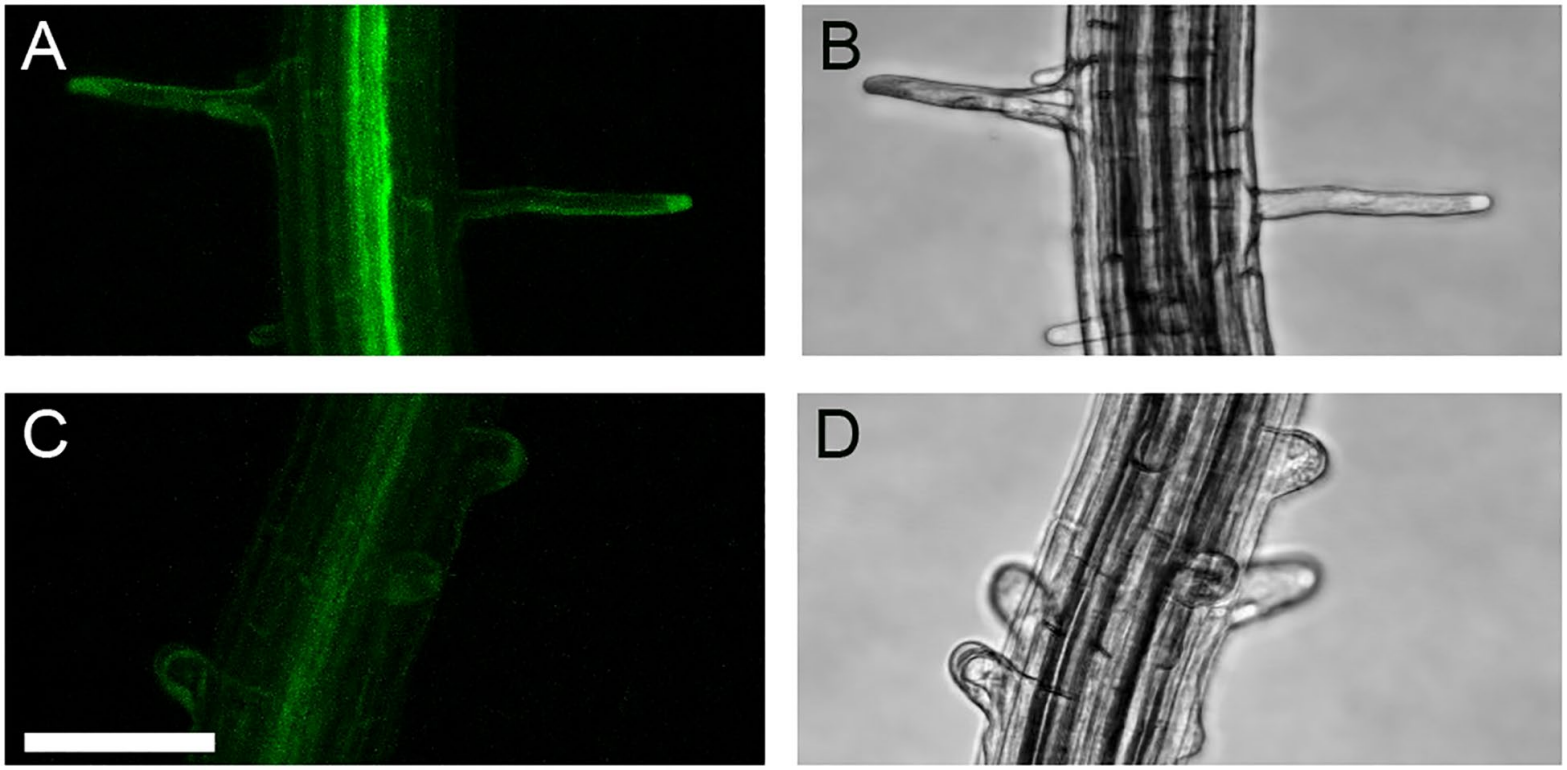
Root hairs of *Atscn1-3* line overexpressing *SCN1* versions. Wild-type *AaSCN1* CDS expression in **(A)** YFP channel and **(B)** transmission channel. Mutant *Aascn1-1* CDS expression in **(C)** YFP channel and **(D)** transmission channel. Scale bar: 75μm.

To confirm that the phenotype observed in the *Aascn1-1* mutant is caused by the mutation in *AaSCN1*, we initially tried rescue experiments in the *A. alpina Pajares scn1-1* mutant but failed to isolate transgenic lines. We therefore performed rescue experiments in *A. thaliana*. We reasoned that the *Atscn1-3* mutant (Carol et al., 2005) may be rescued by wild-type SCN1 from *A. alpina* but not by the *Aascn1-1* mutant protein. Transgenic plants, expressing *A. thaliana* and *A. alpina SCN1* wild-type versions, rescued the root hair defect of *Atscn1-3* in T1 and T2 generations (Figure 7). This indicates that the AaSCN1 protein is fully functional in *A. thaliana*. Transgenic lines, expressing the *Aascn1-1* mutant version, showed a root hair defective phenotype similar to *Atscn1-3* (Figure 7). This indicates that the *Aascn1-1* mutation renders the protein defective and suggests that the mutation in *AaSCN1* causes the root hair branching phenotype.

## Discussion

This study used a forward genetic approach to identify genes involved in root hair morphogenesis and patterning in *A. alpina*. Our phenotypic comparison revealed a similar range of phenotypes as previously described for *A. thaliana* (Grierson and Schiefelbein, 2002). By comparing genes known from *A. thaliana* and those involved in the closely related process of trichome development in *A. alpina*, we uncovered genes affecting most steps of root hair development. Overall, 26 mutant-specific alleles were identified through candidate-driven gene selection and sequencing and whole-genome sequencing including re-sequencing of the respective mutated genes.

Importantly, we correlate mutant-specific alleles with phenotypes. In cases where we found several alleles correlating with the same phenotype, the evidence that the mutations are causal for the phenotype is particularly strong. In cases where we found only one allele, additional proof by rescue experiments is necessary. In these cases, we consider it reasonable to assume that relevant mutations in genes that lead to the same phenotype in *A. alpina* and *A. thaliana* reflect a similar function of these genes in both species.

Our sequence analysis of candidate genes in the respective mutants revealed on average about two mutants for each considered gene. As observed in other screens (Pollock and Larkin, 2004), the distribution of alleles is highly asymmetric, such that we found six gl3 alleles and only one allele for many other genes. However, the average allele frequency of two alleles for each gene suggests that we have identified a representative set of mutants (Chopra et al., 2019).

### Differences and Similarities in Root Hair Patterning Between *A. alpina* and *A. thaliana*

In *A. thaliana*, the R2R3MYB transcription factor WER (Lee and Schiefelbein, 1999) and the WD40 protein TTG1 (Galway et al., 1994) form a complex with the bHLH proteins GL3/EGL3 (Payne et al., 2000; Zhang et al., 2003) that activates the expression of *GL2*, which in turn suppresses root hair development in non-root hair cells (Masucci et al., 1996).

As expected, various mutants with ectopic root hairs showed relevant mutations in *AaTTG1* and *AaGL3*. Similar as had been shown for trichomes (Chopra et al., 2019), *Aagl3* mutants showed a strong phenotype and none of the mutants was defective in *AaEGL3*. We found no mutations in *AaWER*. This is particularly interesting as we have previously found no *gl1* mutants in glabrous trichome mutants (Chopra et al., 2019). While this may be due to the mutant screen not having been fully saturated, it is also possible that the MYB genes act redundantly in *A. alpina*, such that single gene mutations do not lead to a phenotype.

Surprisingly, all six mutants displaying a reduced number of root hairs had no mutations in any of the known *A. thaliana* genes in which mutations lead to fewer root hairs.

### Differences and Similarities in Root Hair Morphology Between *A. alpina* and *A. thaliana*

The analysis of the root hair morphology mutants derived from our screen revealed that various genes, including *ARK1*, *SPI*, *COW*, *PFN1*, *CAP1*, and *KJK*, have similar functions as described in *A. thaliana*. In addition, we found one short root hair mutant with a relevant amino acid substitution in another *AaKJK* family member, the *AaCSLD6* gene. Although the corresponding mutant was not yet described in *A. thaliana*, our finding suggests that *CSLD6* is involved in root hair morphogenesis.

We also found possible differences in the gene-phenotype relationships. While in *A. thaliana* only *lrx1 and the lrx1 lrx2* double mutants show a root hair phenotype (Baumberger et al., 2003), we found that a relevant amino acid exchange in the *AaLRX2* gene correlates with a short root phenotype. This suggests that the relative importance of the *AaLRX1* and *AaLRX2* genes is different in *A. alpina* and *A. thaliana*. Also, unlike in *A. thaliana* (Grierson and Schiefelbein, 2002), we observed short but not swollen root hairs in the *Aakeu* mutant. This suggests that mutations in the same gene lead to slightly different phenotypes in the two species. Similarly, *the1* and *anx1* mutants in *A. thaliana* exhibit collapsed, burst, and short root hairs (Duan et al., 2010), while the corresponding *A. alpina* mutants showed short but intact root hairs.

### Truncation of AaSCN1 Leads to a Multiple Root Hair Phenotype

Our attempt to analyze the function of the *AaSCN1* gene was challenging because we failed to generate transgenic lines in *A. alpina* to prove that the mutant phenotype is indeed caused by the mutation in the gene. However, interspecies experiments using *A. thaliana* provided evidence that the mutation in the *AaSCN1* gene renders it non-functional, suggesting that the *scn1* mutant phenotypes are very similar in the two species. This includes not only the morphological phenotype but also the ROS distribution in the mutants of both species (Carol et al., 2005). Moreover, we showed that YFP fused SCN1 from *A. thaliana* and *A. alpina* localized to the tip of growing root hairs.

### Perspective

In this work, we discovered interesting differences in the gene-to-phenotype relationship between *A. alpina* and *A. thaliana* and their further analysis may help to understand the evolution of the underlying gene regulatory networks. Moreover, we found root hair mutants without relevant SNPs in any of the known *A. thaliana* root hair genes. In principle, it is possible that in these cases, SNPs in the promoter regions cause transcriptional changes of the candidate genes. However, as this was rarely observed in EMS screens before, it is conceivable that the mutant phenotypes can be explained by genetic redundancies or mechanistic differences in root hair formation in *A. alpina*. In either case, the identification of the corresponding genes will likely reveal new players important for root hair development.

## Data Availability Statement

The data sets presented in this study can be found in online repositories. The names of the repository/repositories and accession number(s) can be found at: https://www.ncbi.nlm.nih.gov/, PRJNA745061.

## Author Contributions

MH and MM conceived and designed the analysis. MM and DC collected the data. MM, DC, AS, HS, and LS performed the analysis. MH, LS, and MM wrote the paper. KS, MA, and GC provided access to crucial research components. AS, KS, MA, and GC provided revisions to scientific content of the manuscript. All authors contributed to the article and approved the submitted version.

## Funding

This work was supported by the Deutsche Forschungsgemeinschaft grant HU 497 (MH), the SFB680 (MH), and an International Max Planck Research School fellowship (MM).

## Conflict of Interest

The authors declare that the research was conducted in the absence of any commercial or financial relationships that could be construed as a potential conflict of interest.

## Publisher’s Note

All claims expressed in this article are solely those of the authors and do not necessarily represent those of their affiliated organizations, or those of the publisher, the editors and the reviewers. Any product that may be evaluated in this article, or claim that may be made by its manufacturer, is not guaranteed or endorsed by the publisher.

## Footnotes

^1^ www.arabis-alpina.org

^2^ http://www.ncbi.nlm.nih.gov/

^3^ http://pfam.xfam.org/

^4^ www.arabidopsis.org
