# Supplementary material for: Genetic and Molecular Analysis of Root Hair Development in Arabis alpina
Source: Front Plant Sci. 2021 Oct 15;12:767772. doi: 10.3389/fpls.2021.767772 (PMC8554057; doi:10.3389/fpls.2021.767772)
Supplement: Supplementary file 1 [file Data_Sheet_1.zip › Supplementary Figures.PPTX]

## Slide 1
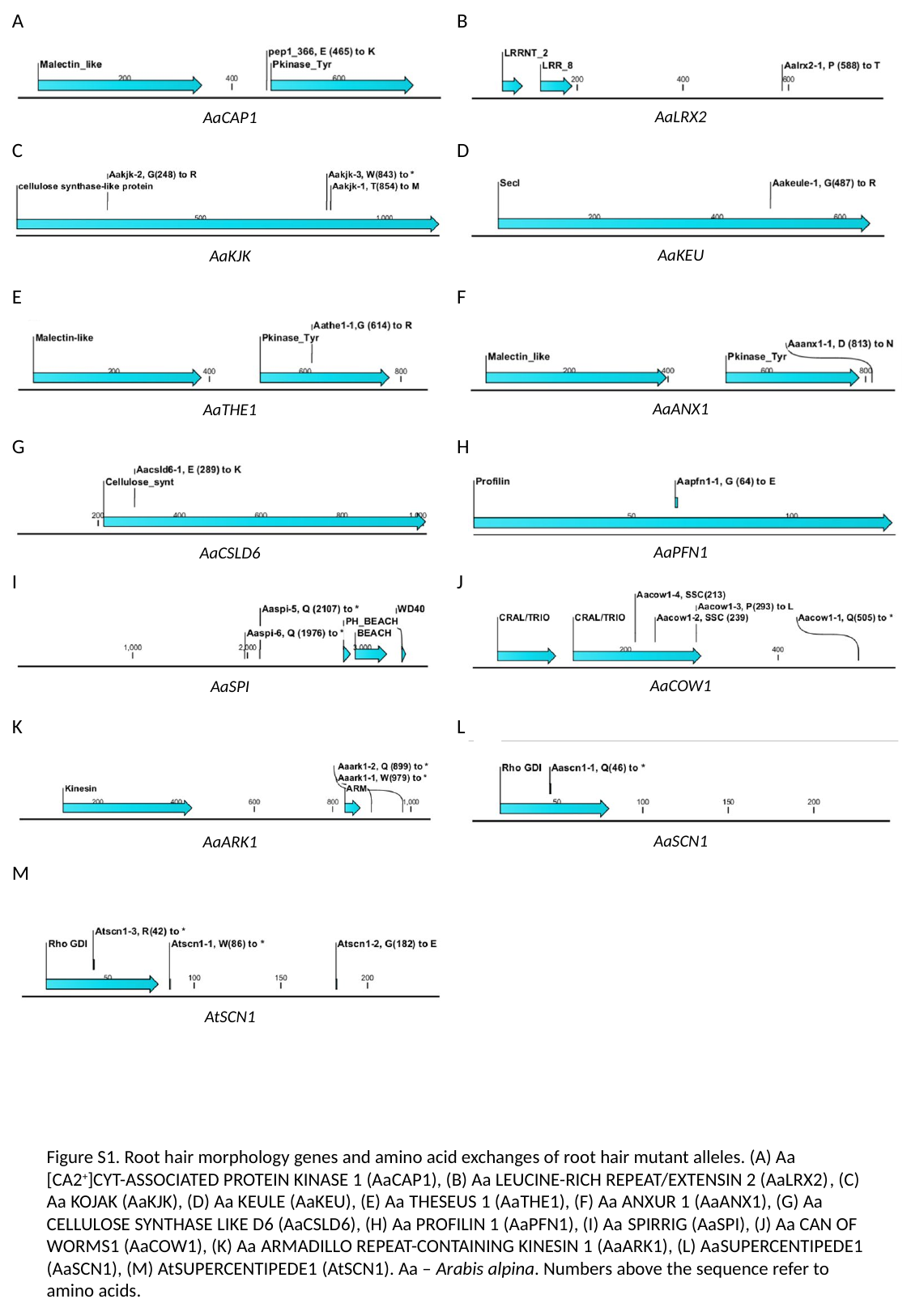

A
B
AaLRX2
AaCAP1
C
D
AaKEU
AaKJK
E
F
AaANX1
AaTHE1
G
H
AaPFN1
AaCSLD6
I
J
AaCOW1
AaSPI
K
L
AaSCN1
AaARK1
M
AtSCN1
Figure S1. Root hair morphology genes and amino acid exchanges of root hair mutant alleles. (A) Aa [CA2+]CYT-ASSOCIATED PROTEIN KINASE 1 (AaCAP1), (B) Aa LEUCINE-RICH REPEAT/EXTENSIN 2 (AaLRX2), (C) Aa KOJAK (AaKJK), (D) Aa KEULE (AaKEU), (E) Aa THESEUS 1 (AaTHE1), (F) Aa ANXUR 1 (AaANX1), (G) Aa CELLULOSE SYNTHASE LIKE D6 (AaCSLD6), (H) Aa PROFILIN 1 (AaPFN1), (I) Aa SPIRRIG (AaSPI), (J) Aa CAN OF WORMS1 (AaCOW1), (K) Aa ARMADILLO REPEAT-CONTAINING KINESIN 1 (AaARK1), (L) AaSUPERCENTIPEDE1 (AaSCN1), (M) AtSUPERCENTIPEDE1 (AtSCN1). Aa – Arabis alpina. Numbers above the sequence refer to amino acids.

## Slide 2
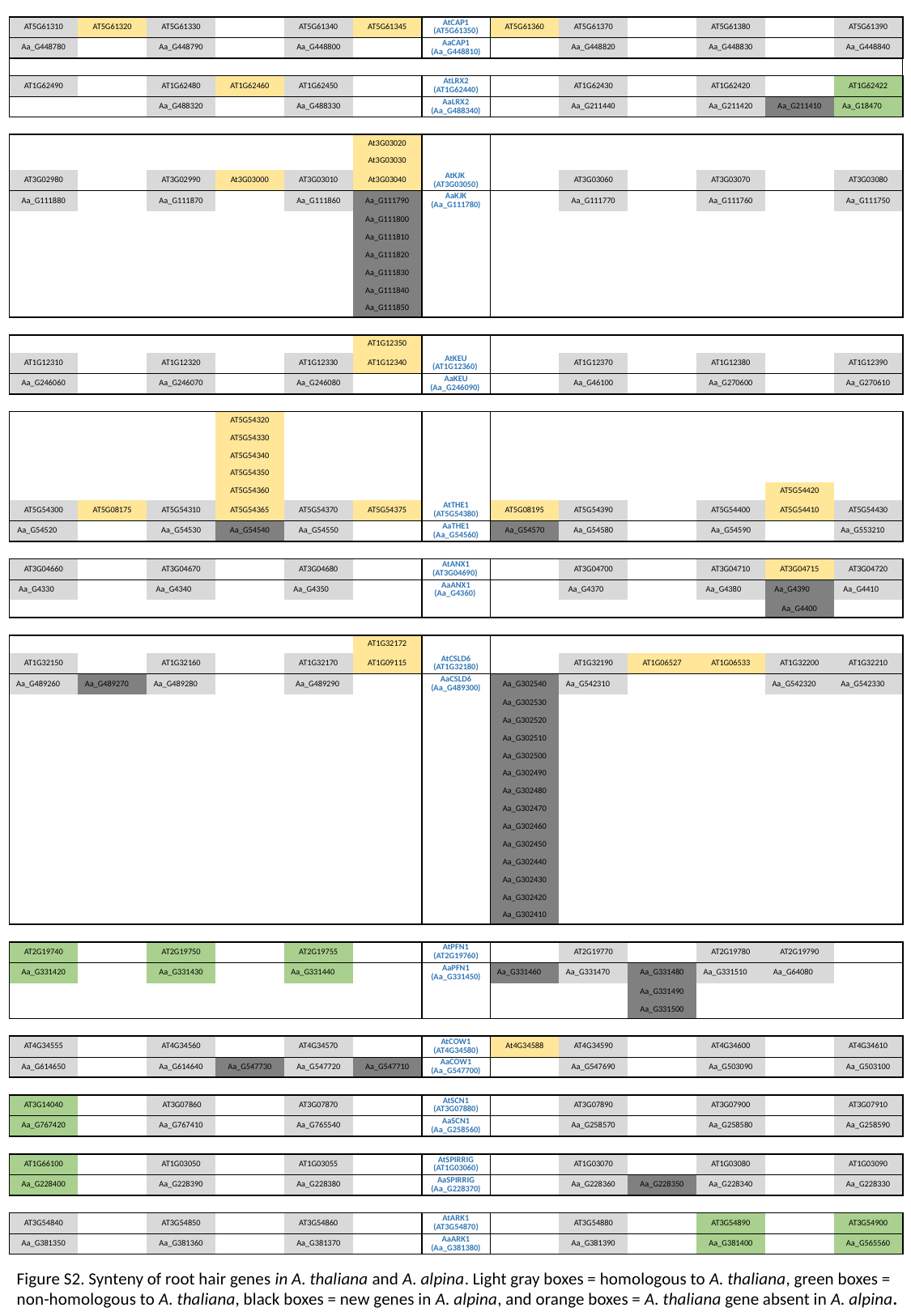

| AT5G61310 | AT5G61320 | AT5G61330 | | AT5G61340 | AT5G61345 | AtCAP1 (AT5G61350) | AT5G61360 | AT5G61370 | | AT5G61380 | | AT5G61390 |
| --- | --- | --- | --- | --- | --- | --- | --- | --- | --- | --- | --- | --- |
| Aa\_G448780 | | Aa\_G448790 | | Aa\_G448800 | | AaCAP1 (Aa\_G448810) | | Aa\_G448820 | | Aa\_G448830 | | Aa\_G448840 |
| | | | | | | | | | | | | |
| AT1G62490 | | AT1G62480 | AT1G62460 | AT1G62450 | | AtLRX2 (AT1G62440) | | AT1G62430 | | AT1G62420 | | AT1G62422 |
| | | Aa\_G488320 | | Aa\_G488330 | | AaLRX2 (Aa\_G488340) | | Aa\_G211440 | | Aa\_G211420 | Aa\_G211410 | Aa\_G18470 |
| | | | | | | | | | | | | |
| | | | | | At3G03020 | | | | | | | |
| | | | | | At3G03030 | | | | | | | |
| AT3G02980 | | AT3G02990 | At3G03000 | AT3G03010 | At3G03040 | AtKJK (AT3G03050) | | AT3G03060 | | AT3G03070 | | AT3G03080 |
| Aa\_G111880 | | Aa\_G111870 | | Aa\_G111860 | Aa\_G111790 | AaKJK (Aa\_G111780) | | Aa\_G111770 | | Aa\_G111760 | | Aa\_G111750 |
| | | | | | Aa\_G111800 | | | | | | | |
| | | | | | Aa\_G111810 | | | | | | | |
| | | | | | Aa\_G111820 | | | | | | | |
| | | | | | Aa\_G111830 | | | | | | | |
| | | | | | Aa\_G111840 | | | | | | | |
| | | | | | Aa\_G111850 | | | | | | | |
| | | | | | | | | | | | | |
| | | | | | AT1G12350 | | | | | | | |
| AT1G12310 | | AT1G12320 | | AT1G12330 | AT1G12340 | AtKEU (AT1G12360) | | AT1G12370 | | AT1G12380 | | AT1G12390 |
| Aa\_G246060 | | Aa\_G246070 | | Aa\_G246080 | | AaKEU (Aa\_G246090) | | Aa\_G46100 | | Aa\_G270600 | | Aa\_G270610 |
| | | | | | | | | | | | | |
| | | | AT5G54320 | | | | | | | | | |
| | | | AT5G54330 | | | | | | | | | |
| | | | AT5G54340 | | | | | | | | | |
| | | | AT5G54350 | | | | | | | | | |
| | | | AT5G54360 | | | | | | | | AT5G54420 | |
| AT5G54300 | AT5G08175 | AT5G54310 | AT5G54365 | AT5G54370 | AT5G54375 | AtTHE1 (AT5G54380) | AT5G08195 | AT5G54390 | | AT5G54400 | AT5G54410 | AT5G54430 |
| Aa\_G54520 | | Aa\_G54530 | Aa\_G54540 | Aa\_G54550 | | AaTHE1 (Aa\_G54560) | Aa\_G54570 | Aa\_G54580 | | Aa\_G54590 | | Aa\_G553210 |
| | | | | | | | | | | | | |
| AT3G04660 | | AT3G04670 | | AT3G04680 | | AtANX1 (AT3G04690) | | AT3G04700 | | AT3G04710 | AT3G04715 | AT3G04720 |
| Aa\_G4330 | | Aa\_G4340 | | Aa\_G4350 | | AaANX1 (Aa\_G4360) | | Aa\_G4370 | | Aa\_G4380 | Aa\_G4390 | Aa\_G4410 |
| | | | | | | | | | | | Aa\_G4400 | |
| | | | | | | | | | | | | |
| | | | | | AT1G32172 | | | | | | | |
| AT1G32150 | | AT1G32160 | | AT1G32170 | AT1G09115 | AtCSLD6 (AT1G32180) | | AT1G32190 | AT1G06527 | AT1G06533 | AT1G32200 | AT1G32210 |
| Aa\_G489260 | Aa\_G489270 | Aa\_G489280 | | Aa\_G489290 | | AaCSLD6 (Aa\_G489300) | Aa\_G302540 | Aa\_G542310 | | | Aa\_G542320 | Aa\_G542330 |
| | | | | | | | Aa\_G302530 | | | | | |
| | | | | | | | Aa\_G302520 | | | | | |
| | | | | | | | Aa\_G302510 | | | | | |
| | | | | | | | Aa\_G302500 | | | | | |
| | | | | | | | Aa\_G302490 | | | | | |
| | | | | | | | Aa\_G302480 | | | | | |
| | | | | | | | Aa\_G302470 | | | | | |
| | | | | | | | Aa\_G302460 | | | | | |
| | | | | | | | Aa\_G302450 | | | | | |
| | | | | | | | Aa\_G302440 | | | | | |
| | | | | | | | Aa\_G302430 | | | | | |
| | | | | | | | Aa\_G302420 | | | | | |
| | | | | | | | Aa\_G302410 | | | | | |
| | | | | | | | | | | | | |
| AT2G19740 | | AT2G19750 | | AT2G19755 | | AtPFN1 (AT2G19760) | | AT2G19770 | | AT2G19780 | AT2G19790 | |
| Aa\_G331420 | | Aa\_G331430 | | Aa\_G331440 | | AaPFN1 (Aa\_G331450) | Aa\_G331460 | Aa\_G331470 | Aa\_G331480 | Aa\_G331510 | Aa\_G64080 | |
| | | | | | | | | | Aa\_G331490 | | | |
| | | | | | | | | | Aa\_G331500 | | | |
| | | | | | | | | | | | | |
| AT4G34555 | | AT4G34560 | | AT4G34570 | | AtCOW1 (AT4G34580) | At4G34588 | AT4G34590 | | AT4G34600 | | AT4G34610 |
| Aa\_G614650 | | Aa\_G614640 | Aa\_G547730 | Aa\_G547720 | Aa\_G547710 | AaCOW1 (Aa\_G547700) | | Aa\_G547690 | | Aa\_G503090 | | Aa\_G503100 |
| | | | | | | | | | | | | |
| AT3G14040 | | AT3G07860 | | AT3G07870 | | AtSCN1 (AT3G07880) | | AT3G07890 | | AT3G07900 | | AT3G07910 |
| Aa\_G767420 | | Aa\_G767410 | | Aa\_G765540 | | AaSCN1 (Aa\_G258560) | | Aa\_G258570 | | Aa\_G258580 | | Aa\_G258590 |
| | | | | | | | | | | | | |
| AT1G66100 | | AT1G03050 | | AT1G03055 | | AtSPIRRIG (AT1G03060) | | AT1G03070 | | AT1G03080 | | AT1G03090 |
| Aa\_G228400 | | Aa\_G228390 | | Aa\_G228380 | | AaSPIRRIG (Aa\_G228370) | | Aa\_G228360 | Aa\_G228350 | Aa\_G228340 | | Aa\_G228330 |
| | | | | | | | | | | | | |
| AT3G54840 | | AT3G54850 | | AT3G54860 | | AtARK1 (AT3G54870) | | AT3G54880 | | AT3G54890 | | AT3G54900 |
| Aa\_G381350 | | Aa\_G381360 | | Aa\_G381370 | | AaARK1 (Aa\_G381380) | | Aa\_G381390 | | Aa\_G381400 | | Aa\_G565560 |
Figure S2. Synteny of root hair genes in A. thaliana and A. alpina. Light gray boxes = homologous to A. thaliana, green boxes = non-homologous to A. thaliana, black boxes = new genes in A. alpina, and orange boxes = A. thaliana gene absent in A. alpina.
